# Starch-Gelatin-Based Scaffolds for Cartilage Defect Repair: An in vitro Study Supporting Its Potential Clinical Use

**DOI:** 10.1177/19476035251407298

**Published:** 2025-12-23

**Authors:** Vukašin Ugrinović, Đorđe Veljović, Tamara Matić, Julijana Stevanović, Per Wretenberg, Mikael Ivarsson, Nenad Andjelkov

**Affiliations:** 1 Innovation Center of the Faculty of Technology and Metallurgy, University of Belgrade, Belgrade, Serbia; 2 Faculty of Technology and Metallurgy, University of Belgrade, Belgrade, Serbia; 3 School of Medical Sciences, Faculty of Medicine and Health, Örebro University, Örebro, Sweden; 4 School of Health Sciences, Faculty of Medicine and Health, Örebro University, Örebro, Sweden; 5 Department of Orthopedics and Sports Medicine, Shanghai United Family Hospital, Shanghai, People’s Republic of China

**Keywords:** matrix-assisted chondrocyte implantation, starch-gelatin, scaffolds, chondrogenesis

## Abstract

**Objective:**

The aim was to investigate starch-gelatin hydrogels as scaffolds for chondrogenesis and compare these with other materials currently in use regarding cell retention and growth.

**Methods:**

Two variants of starch-gelatin-scaffolds and one chitosan-based scaffold were fabricated by casting and freeze-drying. The resulting materials were analyzed with respect to physicochemical and mechanical properties, cut to size, and seeded with human articular chondrocytes. Cell retention and proliferation were evaluated at 1, 14, and 42 days of culturing. Extracellular matrix production was analyzed by histo- and immunohistochemistry. Comparisons were made with that of commercially available hyaluronan- (Hyalofast^®^) and collagen-based (ChondroGide^®^) scaffolds, and synthesized chitosan hydrogels.

**Results:**

The starch-gelatin materials exhibited highly porous structures stabilized by hydrogen bonding, with swelling behavior similar to native cartilage and favorable mechanical handling properties. Despite differences in initial cell retention, all materials except chitosan supported robust cell growth, reaching similar levels after 14 days. No significant changes were observed between 14 and 42 days with the exception of Hyalofast^®^ showing decreased cell number. Chitosan-supported cell growth was more linear over the culture period, but resulted in only half the cell number by day 42 compared with the other materials. Without cells, Hyalofast and one variant of the starch/gelatin hydrogel degraded before day 42. starch/gelatin scaffolds showed collagen I, II, and aggrecan deposition.

**Conclusion:**

Starch-gelatin scaffolds displayed favorable mechanical properties, supported cell growth comparable to commercial scaffolds, and promoted deposition of cartilage-specific extracellular matrix, highlighting their chondrogenic potential

## Introduction

Cartilage defect repair has been the focus of extensive research for many years, resulting in the development of various therapeutic approaches to address this challenging pathology. Most emerging techniques rely on various artificially synthesized scaffolds, yielding variable outcomes regarding cell adhesion, proliferation, and extracellular matrix (ECM) deposition. Many types of biomaterials used as scaffolds for cartilage repair have been reported until the present with various results. The issues like their cost-effectiveness, the quality of the generated repair tissue in terms of hyaline, fibro-hyaline or a fibrous cartilage deposition, hypertrophy or hypotrophy of the newly synthetized graft, integration of the neocartilage to the surrounding tissue, the simplicity of the scaffold delivery during the surgery, the method of scaffold fixation; all these parameters still represent a major concern in the currently available cartilage repair techniques, thus justifying a need for further improvement of the available methods and materials. To be more specific, one study using Hyalofast combined with multipotent stem cells reported magnetic resonance imaging (MRI)-verified defect filling in 71% to 80% of patients, while the remainder exhibited graft hypotrophy.^1^ The same study also reported variable histology results regarding hyaline-like repair in tissue taken from 3 patients and 2 controls. A similar result for cartilage defect filling was reported when using ChondroGide^®^ as scaffold. In that study, combining ChondroGide and autologous adipose tissue graft and adipose mesenchymal stem cells, 77,8% of the patients have shown a complete defect filling, as judged by MRI. No data on histology were reported in this study.^2^ Regarding the repair tissue histology after the use of ChondroGide alone, in the literature better known as autologous matrix-induced chondrogenesis technique (AMIC^®^), a previous study showed a significantly lower content of glycosaminoglycans (GAGs) than a normal hyaline cartilage, as judged by delayed gadolinium-enhanced MRI of cartilage of the ankle joint.^3^ Another study investigating repaired tissue histology after performing AMIC showed only 20% of the normal hyaline-like tissue, although in a very small patient group.^4^ Finally, a study reported superior results both regarding the defect filling and hyaline-like cartilage repair after the chitosan-based gel use, that is, BST-CarGel^®^, when compared with the microfracture alone. This, however, did not reflect on the clinical improvement after 12 months post-surgery.^5^ To summarize, a recent level I study showed no significant short-term improvement in the clinical outcomes when comparing use of scaffolds to a simple microfracture method, although some results are pointing to long-term clinical benefits with the scaffolds use.^6^

Given the ongoing need to improve clinical outcomes in cartilage repair, this study presents, for the first time, the use of starch/gelatin (SG)-based hydrogels as a platform for promoting chondrogenesis. Starch is highly regarded for its hydrophilicity, biocompatibility, non-toxicity, cost-effectiveness, and broad availability, making it an excellent candidate for biomaterials synthesis.^7,8^ However, despite its natural abundance, native starch exhibits limitations such as low dimensional stability, poor gel content, insufficient mechanical properties, and poor processability in final products.^9^ Gelatin, on the other hand, is notable for its affordability, biocompatibility, biodegradability, and chemical resemblance to collagen—the primary structural protein in connective tissues. In addition, it contains Arg-Gly-Asp (RGD)-like sequences that support cell adhesion, proliferation, migration, and differentiation.^10^ Nevertheless, gelatin-based hydrogels often lack adequate mechanical strength and rapid dissolution in aqueous environments under physiological conditions. Blending two or more polymers presents a simple, yet effective strategy for engineering novel materials with enhanced characteristics required for chondrogenesis. This approach leverages the complementary properties of individual polymers, enabling the design of composites with improved mechanical performance, superior biocompatibility, and tunable functionalities suitable for a range of biomedical applications.^11-13^

The aim of this study was to compare the SG hydrogels with chitosan and other clinically used materials in terms of cell retention and proliferation. For this purpose, we included one of the most widely used and commercially available collagen-based scaffolds—ChondroGide (CG)^14,15^ as well as a hyaluronan-based one—Hyalofast (HF). In addition, pure chitosan-based scaffold was fabricated, given the polymer’s ongoing pre-clinical evaluation and its established clinical applications across a range of biomedical fields.^16-19^ The main study hypothesis was that our novel scaffold for cartilage repair has the same or better characteristics in terms of cell retention, cell growth, and scaffold durability, in comparison with commercially available ones approved for clinical use. A comparison of the composition of ECM between the different scaffolds was not addressed in this study. Also, whether the novel scaffolds are chondro-supportive, or chondro-inductive, will be a subject of further investigations.

## Materials and Methods

### Hydrogel fabrication

The hydrogels were fabricated using a blend of natural polymers, gelatin (#G6650; Merck, formerly Sigma-Aldrich, Darmstadt, Germany), and starch (#S4251; Merck), selected for their biocompatibility, biodegradability, and structural properties. All chemicals and reagents used in the study were of analytical grade and used as received. A proprietary formulation process was used to fabricate porous hydrogels. In brief, starch and gelatin were dissolved in water at a 2:1 ratio and blended under controlled conditions to ensure homogeneity. The mixtures were cast into molds and freeze-dried to obtain 3-dimensional porous structures. The freeze-dried samples were subsequently subjected to thermal treatment, with representative samples cured at 160°C (SG160) and 180°C (SG180). These hydrogels were subsequently evaluated *in vitro* and compared with both commercially available materials and a 2% chitosan-based counterpart. The chitosan-based hydrogel was prepared using chitosan (#448877; Merck) through a process involving casting followed by lyophilization.

### Characterization of hydrogels

Morphological investigations were carried out using a field emission scanning electron microscope (FE-SEM, Tescan MIRA 3 XMU) operating at 20 kV to characterize the hydrogel structure. Prior to imaging, the samples were swollen to equilibrium in phosphate-buffered saline (PBS), frozen at −80°C for 1 h, lyophilized, and sputter-coated with gold using a POLARON SC502 sputter coater to minimize electrostatic charging. Lyophilization was performed by transferring the frozen scaffolds into a freeze-dryer (Beta 2–8 LD plus, Martin Christ, GmbH, Germany) and drying at −60°C under a pressure of 0.011 mbar for 24 h.

Fourier transform infrared (FTIR) spectra of the samples were recorded in absorbance mode using a Nicolet™ iS™ 10 FT-IR spectrometer (Thermo Fisher Scientific) equipped with Smart iTR™ attenuated total reflectance accessories, over the range of 400–4000 cm⁻¹, at a resolution of 4 cm⁻¹ with 20 scans. Prior to analysis, the hydrogels were dried and ground into powder.

The equilibrium swelling ratio (ESR) of hydrogels was calculated in PBS using the equation:

(1) $$ESR=\frac{m_{eq}}{m_{0}}$$

where *m_eq_* denotes the weight of the swollen hydrogel at equilibrium, while the *m_0_* represents the weight of the dry hydrogel. The degree of hydrogel degradation was determined gravimetrically in PBS at 37°C, with the medium replaced every 3 days, using the following equation:

(2) $$DD=\frac{m_{0}-m}{m_{0}}$$

where *m* denotes the dry weight after a given degradation period.

In addition, hydrogel degradation was monitored in culture medium, both in the presence and absence of human chondrocytes, over 42 days of cultivation. The degradation endpoint was defined as the time when the hydrogel became visibly fragmented and could no longer maintain structural integrity. The mechanical properties of the hydrogels were evaluated using a Universal Testing Machine AG-Xplus (Shimadzu, Japan) equipped with a 1000 N load cell (force range 0.01-1000 N). Unconfined compression tests were performed on cylindrical specimens (8 mm × 8 mm) up to 100% deformation at a compression rate of 6 mm/min. Contact between the compression plate and the hydrogel was automatically detected using a contact force of 0.02 N. Prior to testing, all hydrogels were swollen to equilibrium in PBS. The compressive modulus was determined as the slope of the stress-strain curve in the linear region between 0% and 10% strain. At least 3 specimens per hydrogel were tested, and mean values ± standard deviations were reported.

### Isolation of Human Articular Chondrocytes

Cartilage was obtained from discarded tissue from patients undergoing total knee arthroplasty due to medial osteoarthritis. Informed consent was obtained from the patients and the study was approved by the Swedish Ethics Review Authority (case #2019-02770). Healthy cartilage was taken from the lateral weight-bearing areas of the knee not affected by osteoarthritis. The tissue was transferred to refrigerator and isolation of chondrocytes commenced within 18 h. Cartilage was cut in 1- to 1.5-mm^3^ pieces and treated with 100 U/ml of type II collagenase (#C6885, from *Clostridium histolyticum*; Merck) in DMEM/F-12 (#10565018; Thermo Fisher Scientific, Waltham, MA) supplemented with 10 µg/ml gentamicin (Merck) and Antibiotic-Antimycotic supplement (#15240062, Thermo Fisher Scientific). The digestion of the cartilage was proceeded for 18 h at 37°C with slow agitation. The enzyme solution was removed after centrifugation at 200 × *g* and by 2 washing steps with DMEM/F-12, antibiotics and 10% fetal bovine serum (FBS; South America CE, Thermo Fisher Scientific). The pellet was resuspended in chondrogenic medium (DMEM/F-12 supplemented with antibiotics), 10% FBS (#10270106; FBS South America CE, Thermo Fisher Scientific), 40 µg/ml proline (#P0380; Merck), 50 µg/ml 2-phospho-l-ascorbic acid (#49752; Merck), 100 nM dexamethasone (#D1756; Merck), insulin-transferrin-selenium (ITS supplement, #I3146; Merck), and 5 ng/ml human recombinant TGF-β1 (Thermo Fisher Scientific). Cells were cultured at 37°C, 5% CO_2_ and passaged by trypsinization (#25200072, Trypsin-EDTA 0.25%; Thermo Fisher Scientific) when reaching near confluence. Chondrocytes were sub-cultured in chondrogenic medium and used for experiments with hydrogels at passage 3-5.

### Culture of Chondrocytes in Hydrogels

**Figure 1.**
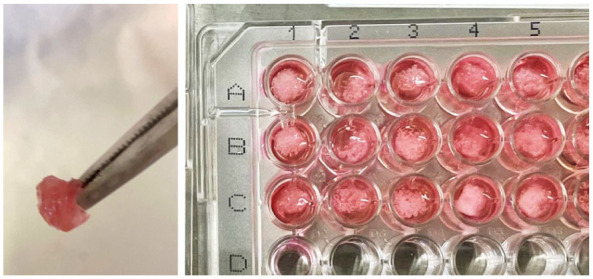
Hydrogels in 96-well plate, equilibrated in chondrogenic culture medium.

The obtained SG and chitosan (CHI) scaffolds, as well as the commercially ones, were cut into 3-mm^3^ pieces to fit into wells of a 96-well plate.The materials were sterilized in 70% ethanol for 30 min. The ethanol was washed out with DMEM/F12 containing 10% FBS and antibiotics for 15 min, repeated twice. The hydrated scaffolds (hereafter referred to as hydrogels) were left overnight to equilibrate with medium. The next day, medium was changed to chondrogenic medium. After 15 min, medium was changed to fresh medium. At passage 3-5, chondrocytes were trypsinized and counted. In total, 40,000 cells in 30-µl chondrogenic medium were seeded into each hydrogel in quadruplicates. The following day (“day1”), hydrogels were transferred to new wells, to separate them from cells that ended up at the bottom of the wells, instead of in the hydrogels (**Fig. 1**). Chondrogenic medium of 150 µl was then added to each hydrogel and culturing proceeded (day 1 cultures were directly incubated with CellTiter-Blue^®^ [CTB] assay as below). Medium was changed twice a week.

### Reference Cells

At day 1, besides seeding cells in hydrogels, 4,000 chondrocytes were seeded in separate wells to act as reference for cell number in the subsequent viability assays.

### Cell Viability Assay

Cell viability was monitored at days 1, 14, and 42 of culture by the CellTiter-Blue Cell Viability Assay (#G8080; Promega Corporation, Madison, WI). Following transfer of the hydrogels to new wells (to avoid interference by cells at the bottom of the wells), 150-µl chondrogenic medium and 30-µl CTB reagent were added to each well. Metabolic reduction was allowed to proceed for 4 h at 37°C and the supernatants stored at −20°C to collect all samples for the fluorescence measurement. When the culture period was over, supernatants with CTB metabolic reduction products were thawed and subjected to fluorescence measurement at 560_Ex_/590_Em_ nm. As reference, CTB supernatants of overnight cultures of 4,000 cells in triplicate were assayed together with gel samples. Fluorescence from culture medium only was used as background control and reduced from the signal generated from samples with cells.

### Confocal Microscopy—Visualization of Cell Proximity to the SG Material

Chondrocytes were cultured in SG160 for 7 days. The cells in hydrogel were labeled with 0.3-µM MitoLite CMXRos (#22698, AAT Bioquest; Nordic Biosite, Stockholm, Sweden) and 2-µM Hoechst 33342 for 30 min. Unbound fluorophores were washed out with two 5-min rinses of PBS and gels fixed with 4% paraformaldehyde for 30 min. Fixative was washed out with two 5-min rinses of PBS, and gels sliced in 0.5-mm sections with a scalpel and mounted onto a glass slide with Fluoromount-G™ Mounting Medium (Thermo Fisher) and a thin string of silicone encircling the gel. Alternatively, cells in hydrogel were permeabilized with 0.1% Triton X-100 post-fixation and stained with Rhodamine Phalloidin Reagent (ab235138; Abcam, Cambridge, UK) diluted 1:500, to visualize F-actin and mounted as described. The gels were then analyzed with confocal microcopy (Olympus IX FluoView FV1000).

### Histochemistry—Visualization of Starch and Gelatin/Collagen

Hydrogels were fixed in 4% paraformaldehyde (#158127; Merck) for 20 min and washed 3 times in PBS pH 7.5. The samples were then dehydrated in increased ethanol concentration, xylene-ethanol, and xylene baths. They were embedded in paraffin (3 consecutive baths), followed by cooling in refrigerator. Paraffin blocks were sectioned to glass slides (#631-9483; Superfrost Plus, VWR International, Radnor, PA) at a thickness of 7 µm, followed by drying and adhesion at 40°C for 1 h. Sections were rehydrated in xylene and ethanol and finally water. To visualize starch and gelatin in the same section, the sections were first incubated in picrosirius red (Direct Red 80 [#365548; Merck] and picric acid) for 1 h, a quick rinse in water followed by Lugol’s Iodine (#PL-7052; Pro-Lab Diagnostics, Richmond Hill, ON, Canada) for 5 min. Sections were then dehydrated over ethanol to xylene and mounted (#3989; Eukitt^®^ Quick hardening mounting medium; Merck).

### Histochemistry—Visualization of ECM and Cell Nuclei

Paraffin-embedded sections were dewaxed and rehydrated as described above. Sections were incubated with 1 µg/ml DAPI (#D1306; Thermo Fisher Scientific) for 5 min followed by eosin Y staining (#152880250; Thermo Fisher Scientific) for 1 min and Lugol’s solution for 5 min. A quick rinse in distilled water was performed between each staining step. Sections were mounted with Fluoromount-G (#00-4958-02; Thermo Fisher Scientific) and examined with fluorescence and light microscopy.

### Immunohistochemistry of Collagen I, II, and Aggrecan

Dewaxing and rehydration of sections were performed as described. Antigen retrieval was performed with mild enzymatic treatment for 15 min at 37°C (0.2 mg/ml pepsin; #P7000; Merck) for collagens and 5 µg/ml proteinase-K (#EO0491; Thermo Fisher Scientific). Stronger proteolytic conditions or heat-induced antigen retrieval resulted in loss of material from the sections. Immunostainings were performed with ABC method, (#ab64264, Mouse and Rabbit Specific HRP/DAB [ABC] Detection IHC kit; Abcam) and antibodies against collagen I (1:400, clone EPR7785, #ab138492; Abcam), collagen II (1:200, #ab34712; Abcam) and aggrecan (1:100, clone BC3, #MA3-16888; Thermo Fisher Scientific). Following the kit instructions, sections were stained in Mayer’s hematoxyline (#01820; Histolab Products AB, Gothenburg, Sweden) for 30 s, rinsed in tap water, dehydrated, and mounted with Eukitt resin. A brown precipitate of DAB indicated positive staining, which was evaluated by light microscopy.

### Statistics

The result from the CTB assay is presented as mean cell number per hydrogel and standard deviation of 4 replicates. Significant difference was calculated using *t*-test with Bonferroni correction or analysis of variance (ANOVA) with Tukey’s post hoc, as indicated. Comparisons with *P* < 0.05 were considered statistical significant.

## Results

### Physico-Chemical, Mechanical Properties, and Stability of the Hydrogels

**Figure 2.**
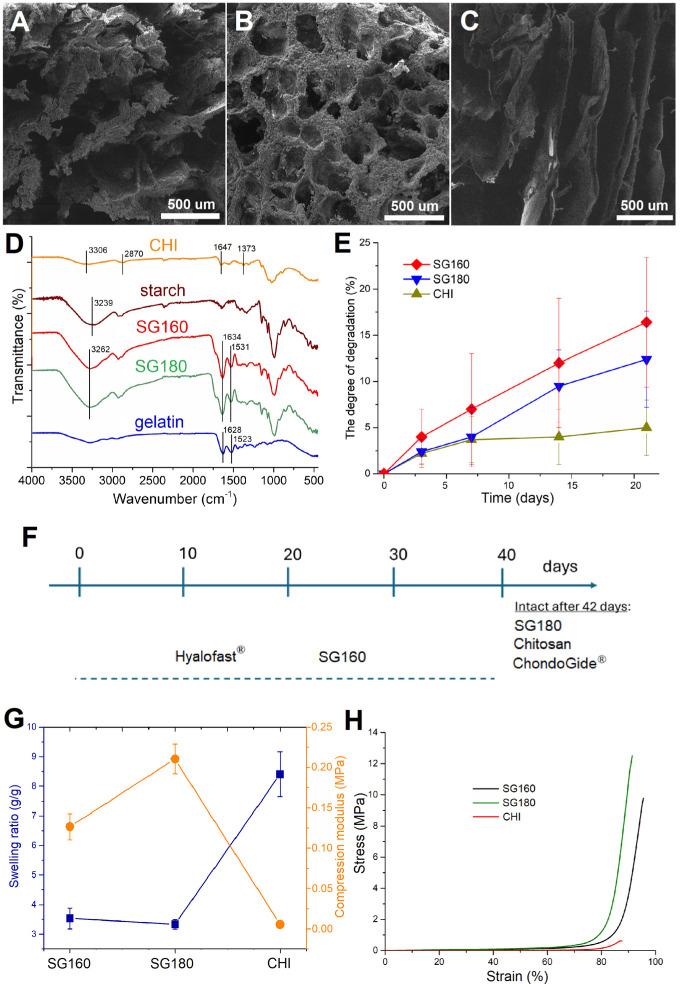
Morphological and mechanical properties of SG and CHI scaffolds. SEM micrographs of the cross-sections of lyophilized hydrogels: (A) SG160, (B) SG180, and (C) CHI. (D) FTIR spectra of pure starch and gelatin, as well as SG and CHI hydrogels. (E) Gravimetrically measured degradation kinetics of the hydrogels in PBS at 37°C. (F) Visually observed degradation of the hydrogels in culture medium without cells, over 42 days at 37°C and 5% CO_2_. (G) Swelling ratio and compression modulus of the obtained hydrogels. (H) Representative stress-strain curves for SG and CHI hydrogels.

To comprehensively evaluate the structure-function relationships of the developed hydrogels, their microstructural organization, chemical composition, stability under simulated body conditions and long-term culture, and mechanical performance were systematically investigated. The combination of starch and gelatin was expected to provide a balance between porosity, bioactivity, and structural integrity, making detailed characterization essential for assessing their suitability in biomedical applications. SEM images (Figures 2A–C) revealed noticeable differences in pore morphology among the hydrogels. SG160 contained irregular pores that reached sizes of about 500 µm, whereas SG180 showed more rounded pores of roughly 300 µm with thicker interconnections. In contrast, the chitosan hydrogel exhibited much higher porosity, characterized by thin necks and elongated lamellar pores that extended up to nearly 1 mm. The FTIR spectrum of chitosan hydrogels revealed several characteristic absorption peaks (**Fig. 2D**). A broad band between 3,291 and 3,361 cm⁻¹ was attributed to overlapping O–H and N–H stretching vibrations, while peaks at 2,916 and 2,870 cm⁻¹ corresponded to symmetric and asymmetric C–H stretching. Distinct amide bands were detected at 1,647 cm⁻¹ (amide I, C=O stretching of residual N-acetyl groups) and 1,550 cm⁻¹ (amide II, N–H bending). In the fingerprint region, a strong peak at 1,373 cm⁻¹ was assigned to CH3 symmetric deformation and C–H bending vibrations with contributions from acetyl groups, whereas a weaker band at 1,310 cm⁻¹ corresponded to amide III (C–N stretching and N–H bending) with possible overlap from saccharide skeletal vibrations.^20^ The FTIR spectrum of gelatin displayed prominent peaks at 1,628, 1,523, and 1,234 cm⁻¹, corresponding to amide I (C=O and C–N stretching of the polypeptide backbone), amide II (N–H bending and C–N stretching), and amide III (C–N stretching and N–H bending), respectively.^21^ For pure starch, characteristic absorption peaks were observed at 1,148 and 991 cm⁻¹, assigned to C–O stretching vibrations of glycosidic linkages. A band near 1,650 cm⁻¹ was associated with H–O–H bending of absorbed water, while a broadband spanning 3650-2970 cm⁻¹ reflected O–H stretching from extensive hydrogen bonding. In addition, a peak around 2930 cm⁻¹ was attributed to C–H stretching.^21,22^ Comparison of the spectra of pure starch and gelatin with those of SG hydrogels confirmed the incorporation of both components. Importantly, characteristic band shifts were observed, indicating molecular interactions between starch and gelatin. The C=O stretching vibration of gelatin’s amide I shifted from 1628 cm⁻¹ to 1634 cm⁻¹, the amide II N–H bending peak shifted from 1523 cm⁻¹ to 1531 cm⁻¹, and the O–H stretching band shifted from 3239 cm⁻¹ in pure starch to 3262 cm⁻¹ in SG hydrogels. These shifts suggest the formation of hydrogen bonding and electrostatic interactions between the 2 polymers, resulting in a modified network structure.

**Figure 3.**
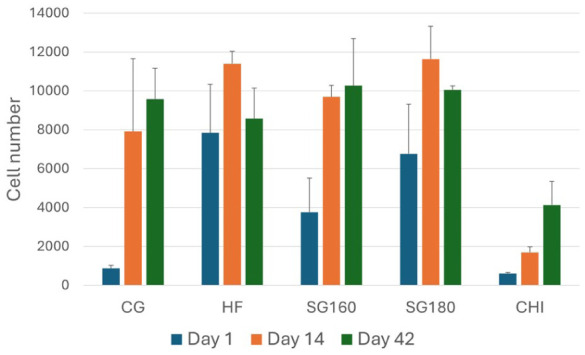
Cell number in the studied scaffolds at 1, 14, and 42 days of culture. Mean cell number and standard deviations are shown (*n* = 4). Statistical significant comparisons between time-points for each scaffold-type are indicated with an asterisk; *P < 0.05; **P < 0.01; ***P < 0.001. CG, ChondroGide; HF, Hyalofast; SG, starch-gelatin; CHI, chitosan.

An important aspect of scaffold properties is stability under physiological conditions. The optimal scaffold should not degrade too quickly, before chondrogenesis begins, nor should it be overly rigid, hindering degradation post-chondrogenesis. While *in vitro* experiments cannot fully predict *in vivo* scaffold performance, they can yield valuable insights into mechanical properties and stability under culture conditions that may influence chondrogenesis. Such data are particularly useful for identifying promising candidates for subsequent *in vivo* studies. Thus, the structural integrity of the hydrogels was evaluated under 2 conditions: (1) in PBS at 37°C, where mass loss was measured gravimetrically over 21 days (**Fig. 2E**), and (2) in cell culture medium, where structural integrity was monitored visually during medium changes throughout the culture period, both in the presence and absence of cells (**Fig. 2F**). It was found that in the case of degradation in PBS both SG samples showed gradual mass loss, with SG160 reaching 16.4% degradation and SG180 showing 12.4% degradation by day 21. In contrast, the CHI hydrogel remained highly stable under the same conditions, with degradation increasing only slightly from 2.2% at day 3 to 5% at day 21. On the other hand, in the cell culture medium, all cell-containing materials (SG, chitosan, Hyalofast, and ChondroGide) were not fragmented over the entire 42 days. This stability was not observed for all scaffolds in absence of cells (**Fig. 2F**): Hyalofast was fragmented after 12 days, SG160 after 26 days while ChondroGide, Chitosan, and SG180 remained relatively intact after 42 days of cultivation. Swelling ratio and mechanical properties of the SG and CHI hydrogels are presented in **Figure 3G and H**. The swelling ratio of the SG hydrogels decreased slightly with increasing curing temperature, from 3.53 ± 0.35 for SG160 to 3.33 ± 0.16 for SG180. In contrast, the CHI hydrogel exhibited a markedly higher swelling ratio of 8.41 ± 0.76. Regarding mechanical properties, the compression modulus of SG hydrogels increased with curing temperature, from 0.127 ± 0.016 MPa for SG160 to 0.211 ± 0.018 MPa for SG180, indicating enhanced stiffness. The CHI hydrogel showed a very low compression modulus of 0.005 ± 0.002 MPa, reflecting its soft and highly swollen structure. These results indicate that cross-linking density and composition strongly influence both the swelling behavior and mechanical strength of the hydrogels.

### Retention of Cells in Scaffolds

An important parameter for successful chondrogenesis is likely the extent to which hydrogels can retain infused cells. To address this, a CTB viability assay was used on day-one hydrogel cultures as described in materials and methods. Retention of cells in this study is defined as the ability of cells to remain in the scaffold 1 day after infusion. The viability assay was performed on hydrogels with gels transferred to a new well, ensuring that no interfering from cells on the bottom of the gel take place. Somewhat surprisingly, variation was quite large regarding capacity of retaining cells in the different hydrogels; SG hydrogels and Hyalofast were most efficient to retaining cells. Cell retention in ChondroGide and chitosan were much less efficient. Based on a statistical comparison, using ANOVA and Tukey’s post hoc, cell retention was decreased in the sequence HF=SG180>SG160>CG=CHI (**Fig. 3**).

### Growth of Cells in Scaffolds

While cell retention is important for evaluating hydrogels suitability for chondrogenesis, cell proliferation is even more critical, as robust ECM deposition depends on it. Thus, to determine growth, cell number was determined at 14 and 42 days in addition to the 1-day point. The first thing that could be concluded from these measurements is that during the first 2 weeks of culture, cell growth was largely inversely correlated to initial cell retention; the only exception being chitosan, showing moderate growth during this period (**Fig. 3**). The second thing was that cell growth was minimal beyond 14 days, as cell number was similar in the different hydrogels at 14 and 42 days. Only in Hyalofast, cell number dropped significantly between these time-points (determined by *t*-test and Bonferroni correction). Chitosan deviated from this by showing a more linear growth over the entire culture period. Taken together, the data indicate that either there was a high retention followed by a limited growth or there was a low retention followed by a more extensive growth. This pattern was not so clear with chitosan, which may be due to a limited maximum cell number that this hydrogel could harbor.

### Preculture Staining of SG Hydrogel Sections

**Figure 4.**
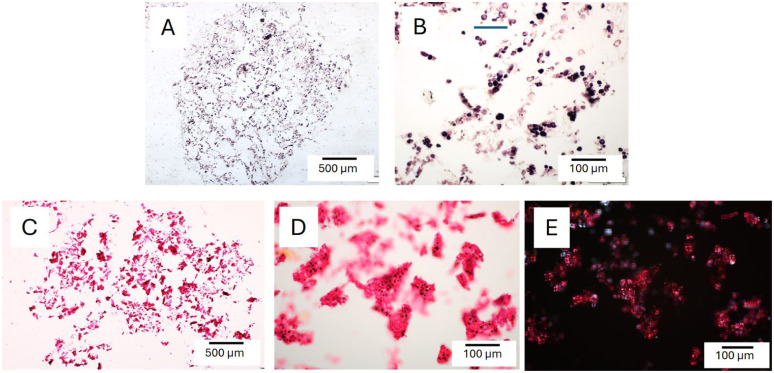
Staining of starch/gelatin hydrogel sections after 1 day in culture medium without cells. Lugol’s solution (dark brown for starch) and picrosirius red (red for gelatin/collagen). (A, B) Sections stained with Lugol’s solution; (C, D) Sections stained with picrosirius red; (E) picrosirius red staining viewed under polarized light.

To visualize the distribution of starch and gelatin components within SG hydrogels, the SG180 hydrogel was processed in the same manner as cell-seeded gels and subsequently stained with Lugol’s solution and picrosirius red. Lugol’s solution alone produced intense staining of the starch component, which exhibited a granular morphology (**Fig. 4A** and **B**). Combined staining with Lugol’s solution and picrosirius red resulted in a strong red coloration, presumably corresponding to the gelatin phase, surrounding dark starch granules. This red signal was also clearly visible under polarized light (**Fig. 4 C-E**).

### Appearance of Hydrogel Sections Stained With Picrosirius Red, Lugol’s Solution and Eosin After 42 Days of Culture

**Figure 5.**
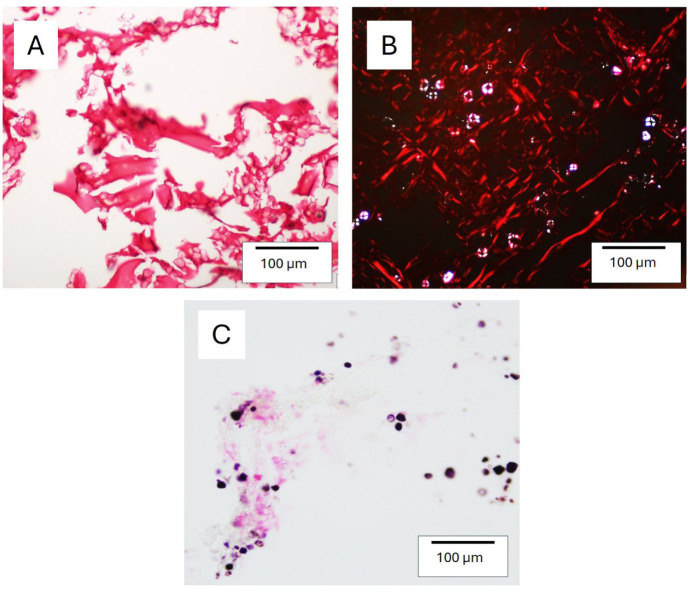
Staining of SG180 hydrogel sections after 42 days of culture using picrosirius red, Lugol’s solution, and eosin. (A) Bright-field image of a section stained with picrosirius red and Lugol’s solution; (B) polarized light image of the same section shown in A; (C) bright-field image of a separate section stained with eosin and Lugol’s solution.

After 42 days of culture, sections of the SG 180 hydrogel stained with picrosirius red and Lugol’s solution showed marked differences compared with preculture samples. The starch component appeared to have been largely degraded or replaced by a more coherent structure that stained positively with picrosirius red. While this staining may indicate either residual gelatin or newly deposited collagen matrix, polarized light imaging revealed the presence of more pronounced fiber-like structures, suggestive of organized collagen. Additional staining with eosin and Lugol’s solution showed limited reaction with the ECM and only sparse residual starch positivity (**Fig. 5**). These observations indicate that cells likely synthesized and deposited collagen, which became organized into fiber-like structures in close association with the gelatin network.

### Attachment of Chondrocytes to the SG Material

**Figure 6.**
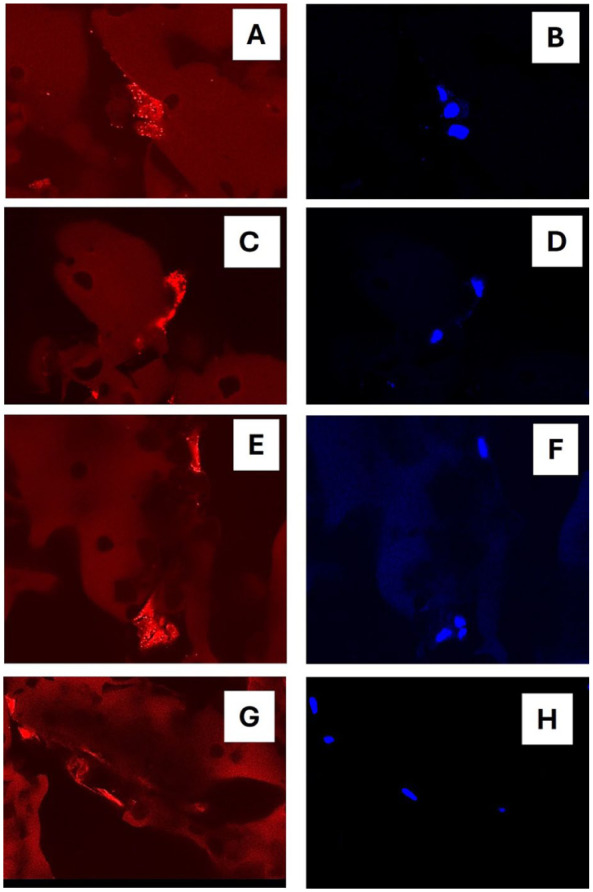
Attachment of chondrocytes to the SG material visualized by confocal microscopy. (A, C, E) Living cells were stained with MitoLite RedCMXRos to visualize active mitochondria. (B, D, F) Nuclear staining with Hoechst 33342 of the same cells. (G, H) Post-fixation staining with rhodamine-phalloidine to visualize actin (red) and nuclear staining of the same cells (blue).

To visualize cellular proximity to the SG scaffold material, chondrocytes were labeled with fluorescent markers for living mitochondria (MitoLite™ Red CMXRos) and DNA (Hoechst 33342) 7 days after initiation of cultures in SG160 (pre-fixation). In addition, cells were labeled with a marker for actin (rhodamine-phalloidin) post-fixation. Confocal microscopy revealed binding or very close proximity of cells to the material which was consistent throughout the hydrogel (**Fig. 6**).

### Cellular Distribution in SG Gels After 42 Days of Culture

**Figure 7.**
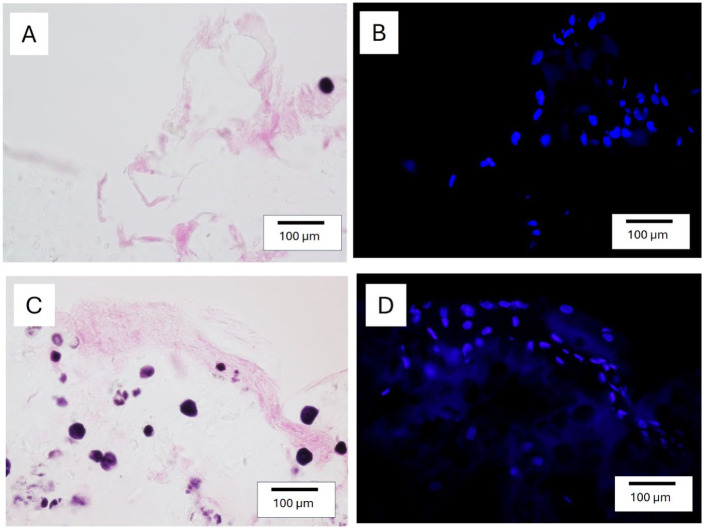
Cell distribution in SG hydrogels cultured with chondrocytes for 42 days. (A, C) Sections stained with eosin to visualize deposited ECM. (B, D) Cell nuclei stained with fluorescent DAPI in same sections as A and C, respectively.

To visualize cells in the SG 180 hydrogel after 42 days of culture, fluorescence DAPI staining was performed in combination with eosin and Lugol’s solution. This approach enabled localization of chondrocytes in relation to areas of cell-derived ECM, since eosin did not stain gelatin in our protocol. Consequently, cells were observed predominantly along eosin-positive areas, which likely corresponded to deposited collagen (**Fig. 7**).

### Immunohistochemistry of ECM Components

**Figure 8.**
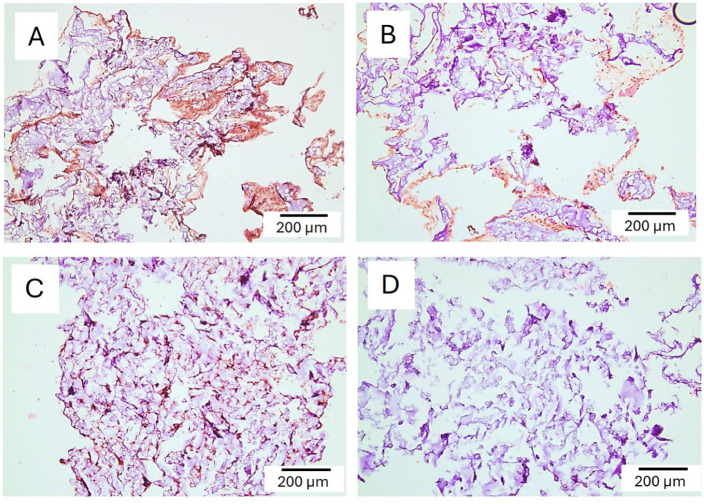
Collagen I, II, and aggrecan expression in SG6 hydrogel cultured with chondrocytes for 6 weeks. (A) Intense broad focal staining of brown DAB precipitate indicating collagen I expression. (B) Focal staining of collagen II expression. Blue color is background staining from hematoxylin. (C) Expression of aggrecan. (D) Negative area of the same hydrogel stained with aggrecan. Magnification is provided with positive signal indicated by an arrow for each antibody.

Next, the presence of specific ECM components within the SG 180 hydrogel was investigated. Collagen I was expressed at a relatively high abundance in the major part of the hydrogels (**Fig. 8A**). Collagen II expression was clearly visible in some areas of the hydrogels, indicating a potential to generate cartilage-specific matrix (**Fig. 8B**). Chondrogenesis is also characterized by expression of proteoglycans, notably aggrecan. Presence of aggrecan was clearly evident in sections of SG6 gels. The staining pattern was, however, quite different from the collagens; while collagens appeared in broad focal areas, aggrecan was more outspread in a more discrete pattern (**Fig. 8C)**. Omitting primary antibodies (negative control) resulted in lack of staining (not shown).

## Discussion

In this study SG-based hydrogels were compared with other natural hydrogels used as scaffolds in clinical approved procedures. While 2 of them (Hyalofast and ChondroGide) are prepared in a solid form,^1,23^ the third one, that is, chitosan-based scaffold, is most often provided in a gel form, at least for cartilage repair, for example, BST-CarGel.^24^ The latter was not included in this study primarily because we wanted all scaffolds to go through the same protocol of preculture procedure, which would not be possible if both solid- and gel-type scaffolds were included.

Each of these scaffolds presents specific advantages and limitations. For instance, chitosan is currently available for clinical use exclusively in a gel formulation (CarGel^®^). This represents an advantage due to its straightforward application via a single intra-articular injection. However, although chitosan may be modified to improve mechanical properties,^25^ its relatively low mechanical strength may limit the capacity to provide sufficient structural support within cartilage. Hyalofast—also sold as a gel formulation—is easy to apply arthroscopically as well, particularly since it is not side-oriented, but exhibits limited mechanical strength, and relatively rapid degradation, which may constrain the time available for tissue ingrowth. We would like to stress that our study is so far performed exclusively under *in vitro* conditions. During (joint) tissue repair, the conditions are more harsh, including the presence of matrix-degrading enzymes released in the joints during the inflammation phase. Thus, for repair processes related to surgery, this degradation would be expected to be even more pronounced.^26^ Regarding ChondroGide, the main disadvantage is probably its bilayer structure, which entails difficulties in placing it properly onto the porous side against the defect when performing a keyhole surgery. Another disadvantage of this scaffold is the cost-effectiveness, and, as mentioned in the introduction section, general issues relating to tissue histology and integration of the newly synthetized tissue with the surrounding cartilage.^2^

Cartilage is a highly complex and anisotropic tissue, characterized by a distinct multilayered structure that imparts exceptional mechanical strength and durability. These intrinsic features make the development of materials suitable for effective cartilage repair particularly challenging. An ideal biomaterial for cartilage repair must exhibit not only adequate mechanical properties, but also good biocompatibility, porosity, lubricity, and the capacity to support chondrocyte function. Increasingly, advanced materials are also expected to incorporate drug delivery functionality for localized treatment of joint pathologies. Although a wide range of materials have been investigated in recent years, few satisfy all the critical requirements for successful clinical application. In this study, we developed cartilage-mimicking hydrogels composed of starch and gelatin, designed specifically for cartilage repair. The native cartilage ECM is primarily composed of type II collagen and proteoglycans enriched with GAG chains.^27^ Type II collagen not only provides tensile strength and structural integrity but also contains bioactive domains that promote cell adhesion, proliferation, and chondrogenic differentiation via integrin-mediated signaling. Proteoglycans, through their negatively charged GAG chains, attract cations and water, thereby increasing tissue osmolarity and contributing to cartilage hydration via the Donnan effect. The high water content of articular cartilage—typically ranging from 65% to 85%—enables the tissue to resist compressive loads through interstitial fluid pressurization, which also supports its low-friction, wear-resistant behavior.^28^

Although our hydrogels did not fully replicate the biochemical composition of native cartilage, the high gelatin content was intended to mimic both the structural and biological roles of type II collagen, while starch served as a polysaccharide analogue to proteoglycans. Together, these components contributed to a highly hydrated, bioactive matrix that could provide a favorable environment for chondrogenesis. SEM analysis revealed that SG 160 hydrogels exhibited irregularly shaped pores, reaching up to ~500 µm, whereas SG180 displayed more rounded pores of ~300 µm in diameter with stronger interconnecting necks. Both SG variants showed lower overall porosity compared with chitosan hydrogels, which was characterized by thin necks and elongated, lamellar pores extending up to ~1 mm. FTIR spectra confirmed the successful incorporation of both starch and gelatin, with characteristic shifts indicative of hydrogen bonding and electrostatic interactions between the polymers, resulting in a stabilized network structure. Swelling experiments further revealed the highly hydrated nature of SG hydrogels, with the ESR values of 3.53 and 3.33 g/g for SG160 and SG180, corresponding to water contents of 71.7% and 70.0%, values falling well within the water content range of the native cartilage.

One of the most important properties of scaffolds used in cartilage repair is their durability and ability to be completely resorbed once implanted in the joint.^29^ By durability, we are referring to the presence of a scaffold in its intact form at the area of repair, giving the necessary mechanical support during the regeneration process. Degradation kinetics is highly relevant; in the most optimal case, a scaffold should be present in the area of repair as long as the process of tissue regeneration has gone so far that it could not be reversed by the absence of the scaffold.^30^ In this study, degradation in PBS at 37°C over 21 days revealed that SG hydrogels underwent gradual mass loss, with SG160 losing 16.4% and SG180 losing 12.4% of their initial mass, whereas chitosan hydrogels remained largely stable, degrading only 2%—5% (**Fig. 3E**). In addition to this, SG160 and SG180 hydrogels demonstrated different degradation profile in cell culturing medium without cells, demonstrating higher degradation stability than Hyalofast, and comparable or lower compared with ChondroGide and CHI. Chitosan-based hydrogels are known to be more stable in physiological conditions due to semi-crystalline structure with strong hydrogen bonding, while in the case of SG hydrogels, this demonstrates that both cross-linking temperature and composition modulate the rate of hydrogel degradation, linking the physicochemical properties such as porosity, swelling, and polymer interactions to scaffold stability. Once the regeneration process has reached the critical point, the scaffold should degrade and be resorbed safely, without triggering foreign body reactions. Thus, a timely non-toxic and non-immunogenic degradation in the course of a few weeks is likely an attractive property of a scaffold for cartilage generation. While this is matter of further studies, we found in this study that presence of chondrocytes was crucial for the long-term hydrogel stability in culture medium (**Fig. 3F**). This effect is most likely attributable to the secretion of ECM components by the cells, which progressively infiltrate and reinforce the hydrogel network. The deposition of collagen and GAGs can act as a secondary matrix, reducing scaffold fragmentation and slowing down hydrolytic degradation. On the other hand, the scaffolds that were intact for 42 days even in absence of cells may serve as promising candidates for cell-free implantation (i.e., supporting endogenous cell recruitment from injured area following implantation). Notably, the degradation of the scaffolds could be also monitored by disappearance of the starch component (**Fig. 6B** and **6C**).

While degradation behavior ensures that scaffolds provide temporary support and are eventually resorbed, their effectiveness during this critical period is equally determined by their mechanical performance, as they must provide sufficient stiffness to withstand compressive forces in the joint while maintaining a hydrated, porous structure conducive to cell infiltration and ECM deposition. In this study, the compression modulus of SG hydrogels was strongly influenced by the annealing temperature. SG160 exhibited a modulus of 0.127 ± 0.016 MPa, whereas SG180 showed a significantly higher modulus of 0.211 ± 0.018 MPa, demonstrating that curing at higher temperature enhances stiffness. This improvement can be attributed to increased cross-linking density and possible reorganization of the polymer chains during annealing, which results in a tighter, more interconnected network. In contrast, the CHI hydrogel exhibited a markedly lower modulus (0.005 ± 0.002 MPa), reflecting its soft and highly swollen structure. The high ESR of CHI (8.41 ± 0.76) indicates substantial water uptake, which increases network plasticization and reduces its ability to resist compressive loading. By comparison, the SG hydrogels displayed much lower swelling ratios (3.53 ± 0.35 for SG160 and 3.33 ± 0.16 for SG180). This moderate swelling contributed to the balance between hydration and mechanical stability in SG hydrogels, as excessive water uptake, such as in the case of CHI, led to mechanical weakening.

Taken together, these results demonstrate that both composition and processing conditions play a decisive role in modulating the mechanical properties of hydrogels. The combination of starch and gelatin provides an opportunity to tune network density, swelling behavior, and compressive stiffness through controlled annealing. Specifically, annealing at higher temperature (180°C) yielded SG hydrogels with mechanical properties closer to the physiological requirements of cartilage tissue, while maintaining a hydration level compatible with chondrocyte function. This tunability highlights the potential of SG systems to be adapted for different cartilage repair strategies, depending on the required balance between stiffness, degradation rate, and bioactivity.

In addition to proper mechanical function, the scaffold must promote cell viability and preserve the chondrogenic phenotype, enabling the sustained production of appropriate ECM components.^31^

Since new tissue formation requires the presence of viable cells—in this case, human articular chondrocytes—our initial focus was on hydrogel’s capacity for cell retention and support of subsequent cellular proliferation.^32^ In addition, a critical parameter was the hydrogel’s ability to facilitate ECM deposition, reflecting its capacity to support chondrogenic differentiation *in vitro*.^33,34^

Initial cell retention varied considerably between the scaffolds tested. While Hyalofast and SG180 showed the highest cell retention at day 1, ChondroGide and chitosan scaffolds presented the lowest level. Several factors could explain these differences, particularly pore size, porosity, and material adhesiveness. For the synthesized hydrogels, a clear correlation between pore characteristics and cell retention was observed (**Fig. 1** and **4**). CHI, with its very large pores (up to ~1 mm) and high porosity, showed the lowest retention, likely due to insufficient structural density for long-term attachment. SG160, characterized by pores up to ~500 µm and lower porosity, achieved higher retention than CHI but was outperformed by SG180. The latter, with smaller pores (~300 µm) and further reduced porosity, supported the highest level of cell retention, underscoring how tighter microstructures can favor sustained cell attachment. In addition, curing conditions were found to influence pore size and stability (**Fig. 3**), highlighting a tunable balance important for optimizing chondrogenesis *in vivo*. Notably, more stringent curing conditions reduced pore size, but increased hydrogels’ stability (**Fig. 3**), highlighting a tunable balance important for optimizing chondrogenesis *in vivo*. In contrast, the commercially available scaffolds showed less straightforward relationships between cell retention and pore size: Hyalofast is reported to have a variable pore size of tens to 100 µm (#651122; Anika Therapeutics, Bedford, MA), and ChondroGide 50-180 µm, tissue side (#30890.3; Geistlich Pharma AG, Wolhusen, Switzerland), both considerably smaller than those of the experimental hydrogels. Despite this, significant differences in cell retention were still evident, suggesting that factors such as surface adhesiveness, for example, mediated by gelatin,^35^ may also play important roles.

The rate of cell growth measured between 1 and 14 days was largely inversely correlated to initial cell retention for all scaffold types except for chitosan. This suggests that a denser structure and/or a pro-mitotic substrate, such as collagen or its analogues, can compensate for low initial retention by promoting subsequent proliferation. Further studies are needed to clarify this phenomenon. Beyond day 14, cell growth ceased in all scaffolds except chitosan, which supported a more linear growth over the entire culture period. For Hyalofast cell number even decreased significantly between days 14 and 42, potentially indicating premature degradation of the scaffold before sufficient ECM had been deposited to maintain structural integrity.^36^ Since our results demonstrate that the SG gels support a robust cell retention and proliferation, as well as ECM deposition, which are the prerequisite for the new cartilage tissue creation, these results are speaking in favor of a potentially useful clinical application of this scaffold in cartilage repair. To confirm chondro-supportive potential of the (SG) hydrogels, one representative formulation (SG6) was further analyzed for expression of cartilage-specific ECM components. This analysis was somewhat hampered by the fact that the nature of the scaffold constituents (carbohydrates and denatured collagen) gave rise to unacceptable background levels when stained by many common chemicals used to detect collagen ECM and proteoglycans. These include picrosirius red, Van Gieson, and aniline blue for collagens, as well as Alcian Blue, Toluidine Blue, Safranin O, and Colloidal Iron for proteoglycans (data not shown). Nevertheless, we found that collagen could be detected by eosin, which did not stain the hydrogel components themselves, and fiber formation was clearly observed with picrosirius red combined with polarized light (**Fig. 7**). Finally, immunohistochemistry was used to detect collagen I, II, and aggrecan after gentle proteolytic treatment of the sections. Although quantitative comparisons between these components are difficult to make (due to demasking efficiency, antibody affinity, etc.), it appears that collagen I dominated over type II, which implicates more fibrocartilage than hyaline cartilage in our model. This may, however, reflect incomplete redifferentiation after several passages of growth on plastic. Aggrecan is an essential component of cartilage, and the presence of this proteoglycan further support chondrogenesis in the hydrogels over the culture period. The major limitation of this study is the fact that it was only performed *in vitro*. But to be eligible for animal studies, proper *in vitro* investigations are needed. This study is aiming to meet, at least in part, this requirements. Thus, we are planning to follow up the present findings by performing animal studies as well. This would show the potential clinical value of this novel scaffold for cartilage repair.

## Conclusion

This study demonstrates that SG-based hydrogels possess several key properties that make them promising candidates for cartilage repair applications. These properties include tunable degradation profiles dependent on thermal curing conditions, effective cell retention and growth comparable to commercially available scaffolds, and ability to support deposition of cartilaginous ECM by chondrocytes. Collectively, these findings indicate that starch/based scaffolds may offer a cost-effective and biologically functional alternative to current commercial products used in cartilage regeneration.
